# Needs for Successful Engagement in Telemedicine Among Rural Older US Veterans and Their Caregivers: Qualitative Study

**DOI:** 10.2196/50507

**Published:** 2024-05-07

**Authors:** Jacqueline Hannah Boudreau, Lauren R Moo, Meaghan A Kennedy, Jennifer Conti, Chitra Anwar, Camilla B Pimentel, Kathryn A Nearing, William W Hung, Eileen M Dryden

**Affiliations:** 1 Center for Healthcare Organization and Implementation Research VA Bedford Healthcare System US Department of Veterans Affairs Bedford, MA United States; 2 New England Geriatric Research, Education, and Clinical Center VA Bedford Healthcare System US Department of Veterans Affairs Bedford, MA United States; 3 Department of Neurology Harvard Medical School Boston, MA United States; 4 Department of Family Medicine Boston University Chobanian & Avedisian School of Medicine Boston, MA United States; 5 Department of Public Health Zuckerberg College of Health Sciences University of Massachusetts Lowell Lowell, MA United States; 6 Eastern Colorado VA Geriatric Research Education and Clinical Center Aurora, CO United States; 7 Division of Geriatric Medicine University of Colorado Anschutz Medical Campus Aurora, CO United States; 8 Bronx Geriatric Research Education and Clinical Center James J Peters VA Medical Center Bronx, New York, NY United States; 9 Department of Geriatrics and Palliative Medicine Icahn School of Medicine New York, NY United States

**Keywords:** caregivers, geriatrics, older adults, rural veterans, rural, specialty care, telehealth, telemedicine, veterans

## Abstract

**Background:**

Telemedicine is an important option for rural older adults who often must travel far distances to clinics or forgo essential care. In 2014, the Geriatric Research, Education, and Clinical Centers (GRECC) of the US Veterans Health Administration (VA) established a national telemedicine network called GRECC Connect. This network increased access to geriatric specialty care for the 1.4 million rural VA-enrolled veterans aged 65 years or older. The use of telemedicine skyrocketed during the COVID-19 pandemic, which disproportionately impacted older adults, exacerbating disparities in specialty care access as overburdened systems shut down in-person services. This surge presented a unique opportunity to study the supports necessary for those who would forgo telemedicine if in-person care were available.

**Objective:**

In spring 2021, we interviewed veterans and their informal caregivers to (1) elicit their experiences attempting to prepare for a video visit with a GRECC Connect geriatric specialist and (2) explore facilitators and barriers to successful engagement in a telemedicine visit.

**Methods:**

We conducted a cross-sectional qualitative evaluation with patients and their caregivers who agreed to participate in at least 1 GRECC Connect telemedicine visit in the previous 3 months. A total of 30 participants from 6 geographically diverse GRECC Connect hub sites agreed to participate. Semistructured interviews were conducted through telephone or the VA’s videoconference platform for home telemedicine visits (VA Video Connect) per participant preference. We observed challenges and, when needed, provided real-time technical support to facilitate VA Video Connect use for interviews. All interviews were recorded with permission and professionally transcribed. A team of 5 researchers experienced in qualitative research analyzed interview transcripts using rapid qualitative analysis.

**Results:**

From 30 participant interviews, we identified the following 4 categories of supports participants described regarding successful engagement in telemedicine, as defined by visit completion, satisfaction, and willingness to engage in telemedicine in the future: (1) caregiver presence to facilitate technology setup and communication; (2) flexibility in visit modality (eg, video from home or a clinic or telephone); (3) technology support (eg, determining device compatibility or providing instruction and on-demand assistance); and (4) assurance of comfort with web-based communication, including orientation to features like closed captioning. Supports were needed at multiple points before the visit, and participants stressed the importance of eliciting the varying needs and preferences of each patient-caregiver dyad. Though many initially agreed to a telemedicine visit because of pandemic-related clinic closures, participants were satisfied with telemedicine and willing to use it for other types of health care visits.

**Conclusions:**

To close gaps in telemedicine use among rural older adults, supports must be tailored to individuals, accounting for technology availability and comfort, as well as availability of and need for caregiver involvement. Comprehensive scaffolding of support starts well before the first telemedicine visit.

## Introduction

Not all older adults have the means or willingness to successfully participate in telemedicine visits. In a survey of medically high-need, high-risk older US veterans, approximately half of respondents were unwilling to engage in video visits. A quarter of those willing lacked the needed technology, and these individuals were more likely to be older, less health literate, or living in more socioeconomically disadvantaged areas [1]. Concerns persist about technology access and use among older adults for telemedicine, such as those who lack broadband access, and common age-associated communication challenges related to sight, hearing, and cognitive limitations [2,3].

However, when telemedicine is successful, its advantages may be especially critical for older, rural adults and their caregivers [4]. Compared to in-person appointments, telemedicine has been shown to have similar, and sometimes better, clinical outcomes [5-8]. It has similar quality, including for visits related to cognitive decline [9], has high levels of patient satisfaction, and is associated with timely access to specialty care [10] which can help support patients with mobility or transportation issues [11,12]. Telemedicine cuts down on travel complications [13], bridging far distances to clinics for older adults living in rural areas [14] and reduces patient and caregiver stress [15]. Further, telemedicine improves care continuity and decreases missed care opportunities [16], including in dementia clinic settings [10].

Those who are older, lower-income, or living in rural areas have historically been less likely to engage in telemedicine visits [17], disparities that worsened during the COVID-19 pandemic. The pandemic disproportionately impacted older adults (World Health Organization) [18], exacerbating disparities in specialty care access as overburdened care systems shut down nonessential in-person services. Even as the overall use of telemedicine soared, gaps among rural patients and those in the oldest age groups widened [19,20]. Yet, the forced pivot to telemedicine provided a unique opportunity to study the support needs of rural, older adults who may not have otherwise used these services.

The US Veterans Health Administration (VA) serves as an optimal setting in which to explore factors influencing successful engagement in telemedicine for rural older patients. More than half of the 2.8 million rural veterans receiving VA services are aged 65 years or older [21]. The VA has been a leading force in telemedicine for 20 years [22,23] in its efforts to expand access to health care services to all veterans, recently celebrating the milestone of 20 years of providing telemedicine services. In 2014, the VA’s Office of Rural Health funded GRECC Connect to use telemedicine to expand access to geriatric specialty care for rural, older veterans with multiple chronic conditions and complex care needs. Geriatric specialists from urban centers of excellence called Geriatric Research, Education, and Clinical Centers (GRECCs) focused on aging worked to build partnerships with rural clinicians to serve older adults living in rural areas through clinic-to-clinic or clinic-to-home visits [24,25].

In spring 2021, we interviewed older adults and their caregivers as part of an evaluation of GRECC Connect’s national network of geriatric telemedicine specialty services. We conducted interviews using the same VA videoconferencing platform used for clinical visits, enabling us to observe and interact with participants as they navigated technological and communication challenges and, if needed, provide necessary technical support or instruction. While other studies have examined satisfaction with telemedicine in older adults [9,10] and competencies of providers, including previsit preparation [26], few studies have looked in-depth at the experiences of older patients inexperienced in telemedicine as they prepare to engage in visits. We sought to (1) elicit patient and caregiver experiences attempting to participate in a video visit with a GRECC Connect geriatric specialist, and (2) explore facilitators and barriers to successful engagement in a telemedicine visit among this group. In this paper, we relay lessons learned from the experiences of these rural older adults and their caregivers, as well as the study team, highlighting the kinds of supports needed for successful engagement in telemedicine among older rural adults and caregivers.

## Methods

### Study Design

We conducted a cross-sectional qualitative evaluation with patients and, when appropriate, their informal caregivers, who had agreed to participate in at least 1 VA GRECC Connect telemedicine visit in the previous 3 months. The study team, led by an anthropologist and health services researcher, was experienced in qualitative research. Throughout the evaluation, the team consulted with an advisory group composed of GRECC Connect leaders, clinicians with expertise in geriatrics, neurology, and primary care, health services researchers, and a veteran consultant. The VA Bedford Healthcare System’s Institutional Review Board determined this evaluation of GRECC Connect services to be nonresearch.

### Setting

In December 2020, we sent letters to clinical leads at 7 of the 15 GRECC Connect hub sites, interdisciplinary geriatric specialty teams who partner with rural community-based outpatient clinics (CBOCs), to assess their willingness to serve as recruitment sites. Potential sites were chosen based on their diverse geographic location across regions of the United States and high volume of telemedicine visits performed during fiscal year 2020.

### Participants

Interviewees were recruited from lists of patients who agreed to participate in a GRECC Connect telemedicine visit at 1 of the 7 hub sites from December 2020 to March 2021. Inclusion criteria included age of ≥65 years and residential rurality (rural-urban commuting area code >1, in line with VA practice). We defined telemedicine as (1) video visits conducted between a remote geriatric specialist and a patient located at home (VA Video Connect; VVC) or at a rural VA CBOC (clinical video telemedicine; CVT) or (2) telephone visits conducted between a remote geriatrics specialist and a patient. Some participants had agreed to a video visit but, for various reasons, only completed a phone call with the clinician. We reviewed the electronic health record to exclude potential participants who passed away or were currently hospitalized or in hospice.

### Recruitment

We sent patients a recruitment letter detailing the goals of the evaluation and inviting them to participate in an interview. Overall, 1 of 3 team members (CA, JHB, and JC) conducted a brief phone screening with the patients and, in some instances, caregivers who agreed to participate to confirm recall of the GRECC Connect index appointment—a recent GRECC Connect telemedicine appointment around which to ground the interview. During the screening call, a staff member administered a technology questionnaire to gauge participant comfort with and access to technology (Multimedia Appendix 1).

### Data Collection

We asked participants about their preference for completing the interview through VVC videoconference or telephone. Members of the study team (CA, JHB, JC, and EMD) conducted semistructured qualitative interviews using participants’ preferred modality. In cases where patient participants had some degree of cognitive impairment (eg, dementia) or communication challenges (uncorrected hearing loss), we interviewed the patient-caregiver dyad. Based on needs identified through the technology questionnaire administered during initial screening phone calls, interviewers sometimes began the interview appointments early to provide instruction for initiating and connecting through the videoconference application. We documented technical support needs in interview notes, which offered additional context for the experiences participants shared. Interview questions were drafted by a subset of the study team, shared with the advisory group for feedback, piloted with 2 different older patients, and then finalized. The guide focused on the support received to engage the dyad in telemedicine, what worked and did not, preferences for medical visit modality, satisfaction, and recommendations for improving the telemedicine experience and available support.

### Analysis

Interviews were audio-recorded and professionally transcribed verbatim. The study team (CA, JHB, JC, EMD, and MAK) analyzed interviews using rapid qualitative analysis [27,28]. Analysts summarized individual interviews using a structured template organized by key conceptual categories or domains. We met regularly to develop consensus about the template domains, which included a priori and emergent domains from the interview guide and content of interviews, respectively. To achieve consensus in the content and application of the template used to organize salient aspects of interview transcripts, 2 evaluators summarized 2 initial transcripts. Other members of the evaluation team reviewed this work. We then summarized 10 additional interviews in pairs, or triads, to maintain consistency in applying the template. The remaining transcripts were summarized individually. The team met to resolve uncertainties and refine content domains as needed. Summary templates were condensed into a single matrix where each row contained data for an individual transcript and each column represented a domain. This matrix allowed us to discern and distill patterns in the data within and across domains. We shared key themes and illustrative quotes with the advisory group, who used their expertise to help interpret the findings.

### Ethical Considerations

The VA Bedford Healthcare System Institutional Review Board determined this work was undertaken to inform VA operations as part of program evaluation and quality improvement activities and was not human subjects research.

## Results

### Participants

We interviewed 30 patients and 26 caregivers who had agreed to participate in at least 1 telemedicine visit in the previous 3 months at 7 geographically diverse GRECC Connect hubs (2 in the Midwest, 1 in the Northeast, 2 in the West, and 2 in the South). Table 1 provides a summary of participant and index-visit characteristics as determined from electronic health record data and interview responses. Patient participants were all male, 93% (28/30) non-Hispanic and White, with a mean age of 76 years. Interviewed caregivers (some had additional caregivers) were most often the patient’s spouse (20/26, 77%) and thus were often older adults themselves. A total of 13% (4/30) of the patients we interviewed did not have a known caregiver. During interviews, sometimes the caregiver was the main respondent in cases where the patient experienced cognitive or communication challenges. Indeed, 87% (26/30) of index telemedicine visits with geriatrics specialists were related to the patient’s cognitive challenges.

### Modality of Index Visit

At least two-thirds of participants’ index appointments (specific, recent GRECC Connect appointments used to identify participants and ground interviews) were conducted using a video modality (Table 1). Some had agreed to a video appointment but either missed it or were unable to initiate it through the home videoconference application. Of these, some visits were performed by phone instead. A total of 90% (27/30) of index visits were not the patient’s first telemedicine encounter, though many had only begun telemedicine visits during the COVID-19 pandemic.

**Table 1:** Characteristics of older, rural veteran- and caregiver-interview participants and their index Veterans Health Administration telemedicine visits between December 2020 and March 2021.

Participant characteristics				Values
**Patients (n=30)**				
	Age (years), mean (SD; range)		76 (6.24; 66-87)	
	Male, n (%)		30 (100)	
	**Race and ethnicity, n (%)**			
		White and non-Hispanic	28 (93)	
		White and Hispanic	1 (3)	
		Unknown	1 (3)	
**Caregivers (n=26), n (%)**				
	**Unpaid caregivers**			
		None	4 (13)	
		1 known caregiver	22 (73)	
		>1 known caregivers	4 (13)	
	**Interviewed caregiver relationship to patient**			
		Spouse	20 (77)	
		Nonspousal significant other	1 (4)	
		Child or child’s spouse	4 (15)	
		Other family member	1 (4)	
**GRECC^a^ connect visit characteristics among participants (n=30), n (%)**				
	**Reason for visit**			
		Initial cognitive impairment	16 (53)	
		Follow-up cognitive impairment	10 (33)	
		Other^b^	4 (13)	
	**Visit modality**			
		Phone	7 (23)	
		VA^c^ video connect (VVC^d^—video to home)	8 (27)	
		Clinical video telehealth (CVT^e^—video to rural clinic)	12 (40)	
		CVT per medical record; patient described phone visit	2 (7)	
		Combination of phone and VVC	1 (3)	
	**First experience with telemedicine**			
		Yes	2 (7)	
		No	27 (90)	
		Unknown or no data	1 (3)	
	**Present in visit**			
		Patient only	7 (23)	
		Patient and caregiver	22 (73)	
		Caregiver only	1 (3)	

^a^GRECC: Geriatric Research, Education, and Clinical Centers.

^b^Falls, physical therapy, medication consultation after stroke, evaluation of sleep hygiene.

^c^VA: US Veterans Health Administration.

^d^VVC: VA Video Connect**.**

^e^CVT: clinical video telemedicine.

### Needed or Desired Supports

Participants described supports they experienced or would recommend before a video visit. They noted that such support should be provided at multiple points between appointment scheduling and the time of the appointment to ensure success. Supports (available, desired, or recommended) fell into the following 4 categories: (1) presence of a caregiver; (2) choice and flexibility in visit modality (eg, video visit or phone); (3) technology support, including assurance of compatible devices, instruction, and provision of on-demand assistance; and (4) assurance of comfort with web-based communication, orientation to features of the videoconferencing platform.

### Presence of a Caregiver

Patients relied on caregivers for assistance with technology and communication, as needed, to successfully participate in telemedicine visits. Among those who successfully initiated the video visit, caregivers were frequently responsible for setting up in-home technology. Ease of connecting, thanks to the presence of a family caregiver, led to favorable views on telemedicine. As per 1 caregiver,

I know that older people that aren’t computer savvy or want to be on the computer. They can get a little intimidated and I know that’s a challenge for some.... I go in and I make sure it’s set up and make sure that he’s connected like we did today with you.... It’s been a very easy process to work with him to work with the telehealth and set it up and to go with it.

However, not all caregivers were comfortable with the technology. One said, “I’ve been known to push the wrong buttons sometimes and cause errors, so. Other than my hesitation or lack of confidence in electronics, there really wasn’t [any issues].”

Further, caregivers often supported the patient in communication, liaising between the patient and the clinician, especially for patients with hearing challenges or cognitive impairment. One patient told us during his video interview, “This is my memory here, over my shoulder,” referring to his spouse. However, caregivers cautioned that the clinician should be cognizant of including the patient in communication rather than just relying on the caregiver.

### Choice and Flexibility in Visit Modality

Participants seldom felt that the telemedicine modality had been a choice. Rather, sometimes telemedicine was the only option available due to pandemic-related clinic closures, and the patient or dyad was informed of visit modality at or after appointment scheduling. Other reasons for telemedicine use and location (home or clinic) included patient or caregiver concern about COVID-19, distance from the specialty clinic, and other logistical concerns. One caregiver said, “We were told it was to be done over the telephone because of COVID-19. [The patient] wasn’t vaccinated then and he had a lot of health issues... Especially breathing issues and stuff so, I don’t think they even wanted like to try to take that attempt with him.”

Some said that having options, when possible, would make them more comfortable with telemedicine. For example, a caregiver recalled that the staff member scheduling the patient’s appointment assured her that telemedicine was appropriate for her father’s evaluation but that alternatives were available if the dyad was unsatisfied with the visit’s quality:

I thought [telemedicine] seemed like a good idea, but I was a little bit uncertain as to how effective it would be for the type of evaluation that needed to be done.... I mentioned it to the person that I spoke to when I scheduled the visit for Dad and she was very clear that if it did not seem to go well, that if it didn’t seem to work well that they would, of course, schedule an in-person visit, so I was fine to go ahead and start with the telehealth visit after she said that.

A patient shared the experience of repeatedly being scheduled for CVT appointments from the rural CBOC, even though he would have preferred to do telemedicine appointments from his home. After several visits, he talked to his doctor to have it changed: “I’d rather do it at home here and talk to [the clinician]. And, you know, finally I just told Dr X, I said, you know, I don’t think I need to have all these nurses standin’ around here listening to our call.”

The telephone enabled additional flexibility for telemedicine encounters. Having the ability to switch from video to the telephone if technical issues arose allowed patients and caregivers to continue the appointments and participants to continue interviews. The phone also afforded caregivers the opportunity to join patients’ visits when they were unable to attend in-person. One caregiver, the patient’s child, who lives in another state, explained, “I wasn’t there, but they call me, or my dad calls me on his cell phone so I can overhear the conversation. They put me on speaker, and we’ve done this twice now and it has been wonderful.”

### Technology Support

Participants stressed that having a positive experience with the technology is critical to successful telemedicine engagement. The ability to connect to video appointments varied, with many experiencing roadblocks such as poor internet connectivity and uncertainty about how to connect to the video software, especially from home. One patient who agreed to try but was unable to complete his visit over video said, “They told me I could try [VVC] and I tried it a couple days before and I still couldn’t do it, so I just got frustrated with it.”

Some participants experienced technology challenges due to incompatibility between their equipment and the VA’s videoconferencing platform. While the VA has the capability to offer free, internet-connected tablets to patients who don’t have their own devices, we found participants at 5 out of the 6 VA hub sites had not been offered a device by anyone in the VA. When the availability of these VA-owned devices was raised in interviews, many showed interest in a VA-issued device. One caregiver remarked, “With the tablet, we could walk outside if it gets, like I can’t [go] outside with my [landline] phone so I just thought like a tablet would be great because we could go sit on the bench.... That would be perfect for him.”

Participants desired more formal instruction before the video appointment date, such as test calls or group education for older adults and caregivers. Some preferred printed and mailed materials, while others found it easier to keep track of digital resources and were overwhelmed by receiving too much written information. Appointment reminders sent through electronic mail were 1 channel participants identified for receiving such instructions. A few recalled receiving in-depth instructions to test audio and video before the appointment. Our experience preparing participants for video interviews corroborated this need. As 1 participant said, “(For) telemedicine, if I know how to get on there and if I know how to, you know, set everything up...like today, my wife was able to set it up because you gave her the instructions, and that was great.” One patient who had struggled to connect to the VA videoconferencing platform before the interview said he wished he had received instruction sooner:

I would just say [to VA employees], “Hey, if you don’t get it to work, tell them to scroll down and hit ‘Start.’” ‘Cause, you know, if I would’ve known that. Of course, they probably figure, “Well, you can see that,” and I mentioned earlier, I’m sure that that’s why nothing ever went through because, you know, I never seen the “Start” ‘til [I received live instruction] today.

We observed that some dyads needed more than 1 round of instruction to support successful engagement. Those who performed test calls typically found it helpful and stressed its importance, but some, like the patient previously mentioned, had challenges with test calls but did not know how to reach anyone for live technical support. One participant recommended, “If [the patient is] not connected with the internet and knows a little bit...there could be somebody provided through the VA that would help them.”

Those who participated in CVT visits from a local rural outpatient clinic benefited from additional technology support from a nurse or technician who was present during setup. Participants who experienced this additional help found it valuable. One patient received hearing aids from the telemedicine nurse. When asked if he would recommend CVT to others, another patient said, “Oh heck yeah! Even the lady setting up stuff like this [is] helpful and everything.”

### Comfort With Web-Based Communication

Participants described and displayed variability in comfort with and preferences for telemedicine visits. For CVT appointments from rural community outpatient clinics, in particular, preferences for having staff in the room during the appointment session varied. In a previous example highlighted earlier in this paper, a patient disliked having extra staff in the room for privacy reasons. Another patient we interviewed had positive feedback about a CVT call where the staff member left after setting up the technology and ensuring that the patient and caregiver were comfortable: “Whoever the person was that, you know, got the doctor on the TV and, you know, made us comfortable. Yeah, it worked very well.... I really liked the fact that she left us—the person that set it up left us alone with the doctor so we had privacy. I liked that part of it.” However, another participant liked having a nurse in the room who could ask the clinician questions on his behalf if he needed help with web-based communication. Another participant was unaware of the optional closed captioning feature of the VA’s videoconferencing platform and recommended that this should be promoted for patients with hearing difficulties.

### “More Than Just COVID-19”

Participants were typically satisfied with their telemedicine experience, and many said they were interested in continuing using telemedicine for at least some appointments. One participant said, “I totally agreed to [the telemedicine visit] and the only reason was because of COVID. But then, after we’d done it a couple of times, it was—it’s so much more than COVID. I mean, I think [the VA] should continue doing it all the time.” Some still preferred or would like to at least keep some in-person appointments.

### “They May Surprise You With What They Know”; A Note on Tailoring Supports

Overall, patients and their caregivers desired options tailored to their preferences and needs. Participants noted that not all older adults have the same technological abilities. One participant acknowledged that “[technology is] a great mystery to some [older adults],” but said they personally had “[not] really hesitated to use what they [the VA] offer tech-wise” and that other older patients “may surprise you with what they know.” Still, not all patients or their caregivers were tech-savvy. Some who formerly felt confident in their technological literacy had lost abilities due to aging or cognitive decline, leading to feelings of frustration or shame. One patient, who engineered military jets before his retirement, explained how he went from being among the top in his technical field to relying on his spouse to set up his computer and the effect it had on his confidence:

I’ve worked all over the world. I’ve worked on the F-18s, the design of it, and worked on all the missiles and all this and I was considered one of the top techs or engineers or directors.... Now, when I had a problem, I would invite my wife on my computer. I could probably work on it, but she’s faster and she does it, you know, better or she’ll say, “Remember, you had to do this,” and I didn’t remember that, so I kind of shied away from it, you know?

## Discussion

### Principal Results

Patient and caregiver participants identified supports critical to the successful initiation of video visits. These included an explanation of options (from local clinic vs from home), instruction and test calls, on-demand technology support, and orientation to video communication norms and features, including sensitivity to privacy preferences. Supports desired spanned multiple points in time between when a patient is offered a telemedicine appointment and the appointment itself, suggesting that ongoing support may be needed to adequately prepare patients and their caregivers.

Our work contributes to the research on telemedicine in several ways. Unlike other studies, we provided an in-depth look at the preappointment preparation experiences of a group of older adults inexperienced in telemedicine. Our methods in this study allowed a uniquely intimate perspective on the telemedicine experiences of our participants. By conducting qualitative interviews using the same technology used in these appointments, we were able to solicit participants’ nuanced accounts of their experiences and recommendations, observe some of the challenges they described, and embody a support role to facilitate engagement in real time. Assisting patients in connecting to the video visit through phone or troubleshooting issues using the chat feature, a previously identified support [29], was often helpful and valued by participants. Table 2 shows recommended strategies to support older telemedicine participants and their caregivers based on patient and caregiver experiences, interviewer observations, and input from our advisory group stakeholders.

Proactively assessing the needs of older adults and their caregivers and discussing available supports is needed. While the VA provides some of the supports participants desired, such as on-demand assistance, closed captioning, and 4G-enabled tablets, many participants were not aware of these resources. System-level interventions may be needed to extend tablet services to more VA patients. A recent evaluation of the tablet program to date found that the VA distributed tablets to more than 7000 patients with access needs in more than 850 inpatient settings, mostly for mental health but increasingly for specialty appointments, though broadband has been a challenge [30]. As in other studies [1,31], we identified a segment of older adults who are interested in participating in telemedicine but who lack the appropriate technology. Offering alternatives to these patients, such as conducting the visit from a local clinic, receiving a clinic-issued device, or suggesting borrowing a device from a family member or friend, may enable engagement in video visits [31]. Patients and their caregivers desired more tailored training for video visit technology use. A study done by Hawley et al [31] showed that trainings for older adults tailored to telemedicine interest level and capability and informed by an initial needs assessment reduce barriers to engagement in video visits.

Patients’ physical and psychological comfort should be addressed to ensure a positive experience that respects patient beneficence and autonomy. Other studies have identified previsit preparation as an important clinician competency domain for video telemedicine with older adult patients, including optimizing the clinician environment for audibility and visibility and identifying who should be included in the visit and their roles in the patient’s care [26]. Our results echo privacy concerns in the telemedicine community regarding the sharing of sensitive information and diagnoses with care partners or others who happen to be in the appointment environment, which in some situations can pose distress to patients or caregivers [32,33]. Mishkin and colleagues [32] recommend multilevel strategies to address these challenges, including system-level patient reminders that encourage patients to ensure privacy before joining appointments, provider discretion in the appropriateness of telemedicine for sensitive appointments, and partnering with local clinics to provide private spaces to see distant providers, as the VA does in its GRECC Connect clinical video visits. Greater attention may be needed to respect patient autonomy by eliciting preferences about the presence of staff and care partners during these appointments.

Social and technological needs are not one-size-fits-all and should be approached with compassion and supportive solutions. This may be especially pertinent for appointments with patients being seen for memory loss and other cognitive challenges whose needs and abilities may be rapidly changing as disease progresses, but is important to consider with all older adults, who have a range of technical aptitude. Studies have shown that clinicians anticipate that older adults will have trouble with remote technology [34]. Yet even within what may appear to be a homogeneous group, we saw variation in what supports were wanted and needed.

Many participants did need help successfully engaging in video visits. Caregivers and staff at CBOCs were key supports for setting up the technology, troubleshooting issues, and getting logged into appointments. Numerous studies have found that the presence of a caregiver facilitates engagement in video visits for older adults, particularly for setting up technology and for patients with dementia [13,14,29,35]. Additionally, we found that participants with hearing or cognitive challenges relied on caregivers for communication with clinicians.

**Table 2:** Recommended strategies to support older telemedicine participants and their caregivers based on patient and caregiver experiences from telemedicine appointments between December 2020 and March 2021, interviewer observations from phone and videoconference interviews, and input from our advisory group stakeholders.

Support	Time period		
	Referral or scheduling	Prior to appointment date	Day of appointment
Presence of a caregiver	Clinician or staff include both patient and caregiver in communication, where possible Clinician or staff identify person(s) who will provide or assist with technology	Identified individuals, including caregiver, provide or help test technology Clinician or staff include both patient and caregiver in appointment communications, where possible	If in-home visit, caregiver initiates videoconference technology Clinician or staff include both patient and caregiver in communication, where possible
Choice and flexibility in visit modality	Clinician or staff explain appointment options Clinician or staff solicit patient or caregiver modality preferences If in-home visit, clinic staff provides contact instructions in case of technology challenges	If in-home visit, clinic staff provides contact instructions in case of technology challenges	Clinician or staff provides backup contact in case modality fails If in-home visit, clinic staff solicit patient or caregiver privacy preferences, when appropriate
Technology support	Clinician or staff consult with patient or dyad about technology comfortability and needs Clinician or staff provide information for technology assistance Clinician or staff encourage test call	Hospital-administered individual or group education Clinic staff provide internet-connected devices Clinic staff ensure test call was successful	Clinic staff assist patient or caregiver with connecting to video platform Clinic staff provide contact for troubleshooting technology challenges
Assurance of comfort with virtual communication	Clinician or staff explains videoconference features	Appointment reminders include information about videoconference features	Clinician or staff explains videoconference features If in-home visit, staff or clinician consults patient or caregiver about communication and privacy needs and preferences

### Limitations

We interviewed patients who agreed to a telemedicine visit and to an interview through phone or video, so there may be unexplored prohibitive barriers for this population among those who declined either. However, many of our participants were not offered in-person visit alternatives because of pandemic closures. Further, while we gained insights into the VVC experience through observation through our interview method, we were not able to observe the CVT experience from a VA clinic. Participants reported far fewer technical challenges in these instances, likely because they had on-site technicians who were responsible for establishing the video connection to the hub site specialists. While our study population reflects the demographics of older, rural veterans and their caregivers, it is important to note that our participants were predominantly White and male and typically had female caregivers who were spouses. Future qualitative studies about the experiences of older, rural adults should explore the social and technological needs of those with different ethnic, racial, gender, or sexual identities, including identity-discordant patient-caregiver dyads. Several studies conducted over the same time frame found that patients who are Black and living in lower-income areas are more likely to engage in telephone visits while patients who are White and living in higher-income areas are more likely to engage in video visits [36-38].

### Conclusions

Telemedicine has the potential to expand access to specialty health care services for rural older adults. Yet widening gaps in use following the COVID-19 pandemic surge show that this population needs more support to engage in telemedicine successfully. Older adults and their caregivers may need ongoing support over various touch points to ensure successful engagement in video telemedicine. Supports are not one-size-fits-all and should instead be tailored to individual older patients, considering their access to and comfort with videoconferencing technology, comfort with the clinician, and availability of and need for caregiver involvement. Technology continues to be a barrier for many, in part due to gaps in broadband access in rural areas. If we do not address these barriers, we are increasing the inequity for this most vulnerable population.

## Appendix

Multimedia Appendix 1 During screening calls for interviews with veterans and caregivers who participated in a telemedicine visit between October 2020 and March 2021, qualitative research team members administered a technology questionnaire to gauge participant comfort with and access to technology. This questionnaire was used to help determine interview modality (telephone or videoconference) and anticipate interview technology challenges.

## Abbreviations

CBOC: community-based outpatient clinics
CVT: clinical video telemedicine
GRECC: Geriatric Research, Education, and Clinical Centers
VA: US Veterans Health Administration
VVC: VA Video Connect

## Data Availability

Data sharing is not applicable to this article as the qualitative interview data contains participants’ sensitive information. More information is available from the corresponding author.

## References

[1] Dang S, Muralidhar K, Li S, Tang F, Mintzer M, Ruiz J, Valencia WM (2022). Gap in willingness and access to video visit use among older high-risk veterans: cross-sectional study. J Med Internet Res.

[2] Thomas-Jacques T, Jamieson T, Shaw J (2021). Telephone, video, equity and access in virtual care. NPJ Digit Med.

[3] Anderson M, Perrin A (2017). Technology use among seniors. Pew Research Center for Internet & Technology.

[4] Dryden EM, Kennedy MA, Conti J, Boudreau JH, Anwar CP, Nearing K, Pimentel CB, Hung WW, Moo LR (2023). Perceived benefits of geriatric specialty telemedicine among rural patients and caregivers. Health Serv Res.

[5] Albritton J, Ortiz A, Wines R, Booth G, DiBello M, Brown S, Gartlehner G, Crotty K (2022). Video teleconferencing for disease prevention, diagnosis, and treatment : a rapid review. Ann Intern Med.

[6] Carotenuto A, Rea R, Traini E, Ricci G, Fasanaro AM, Amenta F (2018). Cognitive assessment of patients with Alzheimer's disease by telemedicine: pilot study. JMIR Ment Health.

[7] Carotenuto A, Traini E, Fasanaro AM, Battineni G, Amenta F (2021). Tele-neuropsychological assessment of Alzheimer's disease. J Pers Med.

[8] Dellifraine JL, Dansky KH (2008). Home-based telehealth: a review and meta-analysis. J Telemed Telecare.

[9] Donelan K, Barreto EA, Sossong S, Michael C, Estrada JJ, Cohen AB, Wozniak J, Schwamm LH (2019). Patient and clinician experiences with telehealth for patient follow-up care. Am J Manag Care.

[10] Azad N, Amos S, Milne K, Power B (2012). Telemedicine in a rural memory disorder clinic-remote management of patients with dementia. Can Geriatr J.

[11] Karlsen C, Ludvigsen MS, Moe CE, Haraldstad K, Thygesen E (2017). Experiences of community-dwelling older adults with the use of telecare in home care services: a qualitative systematic review. JBI Database System Rev Implement Rep.

[12] Charness N (2014). Utilizing technology to improve older adult health. Occup Ther Health Care.

[13] Elbaz S, Cinalioglu K, Sekhon K, Gruber J, Rigas C, Bodenstein K, Naghi K, Lavin P, Greenway KT, Vahia I, Rej S, Sekhon H (2021). A systematic review of telemedicine for older adults with dementia during COVID-19: an alternative to in-person health services?. Front Neurol.

[14] Castanho TC, Sousa N, Santos NC (2017). When new technology is an answer for old problems: the use of videoconferencing in cognitive aging assessment. J Alzheimers Dis Rep.

[15] Gately ME, Tickle-Degnen L, McLaren JE, Ward N, Ladin K, Moo LR (2022). Factors influencing barriers and facilitators to in-home video telehealth for dementia management. Clin Gerontol.

[16] Jacobs JC, Blonigen DM, Kimerling R, Slightam C, Gregory AJ, Gurmessa T, Zulman DM (2019). Increasing mental health care access, continuity, and efficiency for veterans through telehealth with video tablets. Psychiatr Serv.

[17] Poeran J, Cho LD, Wilson L, Zhong H, Mazumdar M, Liu J, Memtsoudis SG (2021). Pre-existing disparities and potential implications for the rapid expansion of telemedicine in response to the coronavirus disease 2019 pandemic. Med Care.

[18] Older people and COVID-19. World Health Organization.

[19] Padala KP, Wilson KB, Gauss CH, Stovall JD, Padala PR (2020). VA video connect for clinical care in older adults in a rural state during the COVID-19 pandemic: cross-sectional study. J Med Internet Res.

[20] Hsiao V, Chandereng T, Lankton RL, Huebner JA, Baltus JJ, Flood GE, Dean SM, Tevaarwerk AJ, Schneider DF (2021). Disparities in telemedicine access: a cross-sectional study of a newly established infrastructure during the COVID-19 pandemic. Appl Clin Inform.

[21] Bowser LN, Washington DL (2019). Access to care among rural veterans. Office of Rural Health, Department of Veterans Affairs.

[22] Der-Martirosian C (2020). Spotlight on Telehealth. Health Services Research & Development, U.S. Department of Veterans Affairs.

[23] Lutes T (2023). VA Telehealth Services celebrates 20 years. Department of Veterans Affairs Office of Connected Health.

[24] Pimentel CB, Gately M, Barczi SR, Boockvar KS, Bowman EH, Caprio TV, Colón-Emeric CS, Dang S, Espinoza SE, Garner KK, Griffiths PC, Howe JL, Lum HD, Markland AD, Rossi MI, Thielke SM, Valencia-Rodrigo WM, Moo LR, Hung WW (2019). GRECC connect: geriatrics telehealth to empower health care providers and improve management of older veterans in rural communities. Fed Pract.

[25] Supiano MA, Alessi C, Chernoff R, Goldberg A, Morley JE, Schmader KE, Shay K (2012). Department of Veterans Affairs Geriatric Research, Education and Clinical Centers: translating aging research into clinical geriatrics. J Am Geriatr Soc.

[26] Powers BB, Van Zuilen RM, Schwartz AW, Dang S, McLaren JE, Hoang-Gia D, Moo LR (2023). Competencies for video telemedicine with older adult patients. J Am Geriatr Soc.

[27] Hamilton AB, Finley EP (2020). Reprint of: qualitative methods in implementation research: an introduction. Psychiatry Res.

[28] Gale RC, Wu J, Erhardt T, Bounthavong M, Reardon CM, Damschroder LJ, Midboe AM (2019). Comparison of rapid vs in-depth qualitative analytic methods from a process evaluation of academic detailing in the Veterans Health Administration. Implement Sci.

[29] Dewar S, Lee PG, Suh TT, Min L (2020). Uptake of virtual visits in a geriatric primary care clinic during the COVID-19 pandemic. J Am Geriatr Soc.

[30] Zulman DM QUERI Evaluation of video telehealth tablets. VA Quality Enhancement Research Initiative (QUERI), U.S. Department of Veterans Affairs.

[31] Hawley CE, Genovese N, Owsiany MT, Triantafylidis LK, Moo LR, Linsky AM, Sullivan JL, Paik JM (2020). Rapid integration of home telehealth visits amidst COVID-19: what do older adults need to succeed?. J Am Geriatr Soc.

[32] Mishkin AD, Zabinski JS, Holt G, Appelbaum PS (2023). Ensuring privacy in telemedicine: ethical and clinical challenges. J Telemed Telecare.

[33] Ahmad A, Mustafa A, Qureshi AA, Naseem A, Rana AR, Akhtar M, Rasool A, Sadat U (2024). Telemedicine practice: current challenges of consent and autonomy, patient privacy and data security worldwide. J Soc Prev Advocacy Res KEMU.

[34] Nápoles AM, Appelle N, Kalkhoran S, Vijayaraghavan M, Alvarado N, Satterfield J (2016). Perceptions of clinicians and staff about the use of digital technology in primary care: qualitative interviews prior to implementation of a computer-facilitated 5As intervention. BMC Med Inform Decis Mak.

[35] Cimperman M, Brenčič MM, Trkman P, de Leonni Stanonik M (2013). Older adults' perceptions of home telehealth services. Telemed J E Health.

[36] Rodriguez JA, Betancourt JR, Sequist TD, Ganguli I (2021). Differences in the use of telephone and video telemedicine visits during the COVID-19 pandemic. Am J Manag Care.

[37] Schifeling CH, Shanbhag P, Johnson A, Atwater RC, Koljack C, Parnes BL, Vejar MM, Farro SA, Phimphasone-Brady P, Lum HD (2020). Disparities in video and telephone visits among older adults during the COVID-19 pandemic: cross-sectional analysis. JMIR Aging.

[38] Eberly LA, Kallan MJ, Julien HM, Haynes N, Khatana SAM, Nathan AS, Snider C, Chokshi NP, Eneanya ND, Takvorian SU, Anastos-Wallen R, Chaiyachati K, Ambrose M, O'Quinn R, Seigerman M, Goldberg LR, Leri D, Choi K, Gitelman Y, Kolansky DM, Cappola TP, Ferrari VA, Hanson CW, Deleener ME, Adusumalli S (2020). Patient characteristics associated with telemedicine access for primary and specialty ambulatory care during the COVID-19 pandemic. JAMA Netw Open.

