# Supplementary material for: Needs for Successful Engagement in Telemedicine Among Rural Older US Veterans and Their Caregivers: Qualitative Study
Source: JMIR Form Res. 2024 May 7;8:e50507. doi: 10.2196/50507 (PMC11109863; doi:10.2196/50507)
Supplement: Multimedia Appendix 1 [file formative_v8i1e50507_app1.docx]

## **Appendix A: Technology Questionnaire**

During screening calls, staff members administered a technology questionnaire to gauge participant comfort with and access to technology.

### **Technology Questions: Veteran**

In general, how comfortable would you say you are using technology to communicate?

☐ Not at all comfortable

☐ Somewhat comfortable

☐ Very comfortable

What devices do you have at home? By devices I mean pieces of electronic equipment that you use to get information or communicate with others. Do you have a…

☐ Smart phone (cell phone, iPhone, etc.)?

☐ A tablet (iPad, Amazon Fire, etc.)?

☐ Laptop computer (portable computer)?

☐ Desktop computer?

☐ Any other devices you use?

Which of these do you regularly use – and by that I mean at least once a week?

☐ A smart phone (cell phone, iPhone, etc.)?

☐ A tablet (iPad, Amazon Fire, etc.)?

☐ Laptop computer (portable computer)?

☐ Desktop computer?

☐ Any other devices you use?

Were any of these given to you by the VA (particularly a tablet)?

☐ Yes

☐ No

Do you usually need help when you use these devices?

☐ Yes

Who helps you?

☐ No

How reliable is your internet service?

☐ Unreliable

☐ Fairly reliable

☐ Very reliable

☐ I don’t know

Do you use My HealtheVet?

☐ Yes

☐ No

### **Technology Questions: Caregiver**

In general, how comfortable would you say the Veteran you help is with using technology to communicate?

☐ Not at all comfortable

☐ Somewhat comfortable

☐ Very comfortable

What devices do they have at home? By devices I mean pieces of electronic equipment used to get information or communicate with others. Do they have a…

☐ A smart phone available to them (cell phone, iPhone, etc.)?

☐ A tablet (iPad, Amazon Fire, etc.)?

☐ Laptop computer (portable computer)?

☐ Desktop computer?

☐ Any other devices you use?

Which of these do they regularly use – and by that I mean at least once a week?

☐ A smart phone (cell phone, iPhone, etc.)?

☐ A tablet (iPad, Amazon Fire, etc.)?

☐ Laptop computer (portable computer)?

☐ Desktop computer?

☐ Any other devices you use?

Were any of these given to them by the VA (particularly a tablet)?

☐ Yes

☐ No

Do they typically need help when operating these devices?

☐ Yes

Who helps them?

[If not the CG] Would that person possibly be available to assist with this phone interview?

☐ No

How reliable is their internet service?

☐ Unreliable

☐ Fairly reliable

☐ Very reliable

☐ I don’t know

Does your Veteran use My HealtheVet?

☐ Yes

☐ No

Based on what you’ve shared with me, I’d like to set up our interview using [phone or VVC]. [Record modality.]
